# Lifestyle factors associated with inflammatory bowel disease: data from the Swiss IBD cohort study

**DOI:** 10.1186/s12876-023-02692-9

**Published:** 2023-03-12

**Authors:** Severin A. Lautenschlager, Mamadou Pathé Barry, Gerhard Rogler, Luc Biedermann, Philipp Schreiner, Alexander R. Siebenhüner, Swiss IBD Cohort Study Group, Karim Abdelrahman, Gentiana Ademi, Patrick Aepli, Amman Thomas, Claudia Anderegg, Anca-Teodora Antonino, Eva Archanioti, Eviano Arrigoni, Diana Bakker de Jong, Bruno Balsiger, Polat Bastürk, Peter Bauerfeind, Andrea Becocci, Dominique Belli, José M. Bengoa, Janek Binek, Mirjam Blattmann, Stephan Boehm, Tujana Boldanova, Jan Borovicka, Christian P. BellBraeggeri, Stephan Brand, Lukas Brügger, Simon Brunner, Patrick Bühr, Bernard Burnand, Sabine Burk, Emanuel Burri, Sophie Buyse, Dahlia-Thao Cao, Ove Carstens, Dahlia-Thao Cao, Dominique H. Criblez, Sophie Cunningham, Fabrizia D’Angelo, Philippe de Saussure, Lukas Degen, Joakim Delarive, Christopher Doerig, Barbara Dora, Susan Drerup, Mara Egger, Ali El-Wafa, Matthias Engelmann, Jessica Ezri, Christian Felley, Markus Fliegner, Nicolas Fournier, Montserrat Fraga, Yannick Franc, Pascal Frei, Remus Frei, Michael Fried, Florian Froehlich, Raoul Ivano Furlano, Luca Garzoni, Martin Geyer, Laurent Girard, Marc Girardin, Delphine Golay, Ignaz Good, Ulrike Graf Bigler, Beat Gysi, Johannes Haarer, Marcel Halama, Janine Haldemann, Pius Heer, Benjamin Heimgartner, Beat Helbling, Peter Hengstler, Denise Herzog, Cyrill Hess, Roxane Hessler, Klaas Heyland, Thomas Hinterleitner, Claudia Hirschi, Petr Hruz, Pascal Juillerat, Carolina Khalid-de Bakker, Stephan Kayser, Céline Keller, Christina Knellwolf-Grieger, Christoph Knoblauch, Henrik Köhler, Rebekka Koller, Claudia Krieger-Grübel, Patrizia Künzler, Rachel Kusche, Frank Serge Lehmann, Andrew Macpherson, Michel H. Maillard, Michael Manz, Astrid Marot, Rémy Meier, Christa Meyenberger, Pamela Meyer, Pierre Michetti, Benjamin Misselwitz, Patrick Mosler, Christian Mottet, Christoph Müller, Beat Müllhaupt, Leilla Musso, Michaela Neagu, Cristina Nichita, Jan Niess, Andreas Nydegger, Nicole Obialo, Diana Ollo, Cassandra Oropesa, Ulrich Peter, Daniel Peternac, Laetitia Marie Petit, Valérie Pittet, Rachel Kusche, Daniel Pohl, Marc Porzner, Claudia Preissler, Nadia Raschle, Ronald Rentsch, Alexandre Restellini, Sophie Restellini, Jean-Pierre Richterich, Frederic Ris, Branislav Risti, Marc Alain Ritz, Nina Röhrich, Jean-Benoît Rossel, Vanessa Rueger, Monica Rusticeanu, Markus Sagmeister, Gaby Saner, Bernhard Sauter, Mikael Sawatzki, Michael Scharl, Martin Schelling, Susanne Schibli, Hugo Schlauri, Dominique Schluckebier, Daniela Schmid, Sybille Schmid-Uebelhart, Jean-François Schnegg, Alain Schoepfer, Vivianne Seematter, Frank Seibold, Mariam Seirafi, Gian-Marco Semadeni, Arne Senning, Christiane Sokollik, Joachim Sommer, Johannes Spalinger, Holger Spangenberger, Philippe Stadler, Peter Staub, Dominic Staudenmann, Volker Stenz, Michael Steuerwald, Alex Straumann, Bruno Strebel, Andreas Stulz, Michael Sulz, Aurora Tatu, Michela Tempia-Caliera, Joël Thorens, Kaspar Truninger, Radu Tutuian, Patrick Urfer, Stephan Vavricka, Francesco Viani, Jürg Vögtlin, Roland Von Känel, Dominique Vouillamoz, Rachel Vulliamy, Paul Wiesel, Reiner Wiest, Stefanie Wöhrle, Samuel Zamora, Silvan Zander, Tina Wylie, Jonas Zeitz, Dorothee Zimmermann

**Affiliations:** 1 Department of Gastroenterology and Hepatology, University Hospital Zurich, University of Zurich, Raemistrasse 100, 8091 Zurich, Switzerland; 2 Institute of Social and Preventive Medicine, Université de Lausanne, Lausanne, Switzerland; 3 Department of Medical Oncology, Center for Haematology and Oncology, University Hospital Zurich, University of Zurich, Zurich, Switzerland

**Keywords:** Inflammatory bowel disease, Environmental factors, Swiss IBD cohort study, Nutrition, Physical activity

## Abstract

**Background:**

Various environmental risk factors have been associated with the pathogenesis of inflammatory bowel disease. In this study we aimed to identify lifestyle factors that affect the onset of Crohn’s disease and ulcerative colitis.

**Methods:**

2294 patients from the Swiss IBD Cohort Study received a questionnaire regarding physical activity, nutritional habits and status of weight. In addition, a control group was formed comprising patients’ childhood friends, who grew up in a similar environment.

**Results:**

Overall, 1111 questionnaires were returned (response rate: 48.4%). Significantly more patients with inflammatory bowel disease reported no regular practice of sport during childhood and beginning of adulthood compared to the control group (*p* = 0.0001). No association between intake of refined sugar and onset of inflammatory bowel disease was observed. More patients with Crohn’s disease compared to ulcerative colitis and controls suffered from overweight during childhood (12.8% vs. 7.7% and 9.7%, respectively; *p* = 0.027).

**Conclusions:**

Our study underlines the relevance of environmental factors in the development of inflammatory bowel disease. Our results imply a protective effect of physical activity regarding the onset of inflammatory bowel disease.

## Introduction

Inflammatory bowel disease (IBD) is a chronic and relapsing inflammatory disorder of the gastrointestinal tract and includes the main subtypes Crohn’s disease (CD) and ulcerative colitis (UC). Incidence and prevalence of IBD in Western countries have been on the rise since the beginning of the twentieth century [1]. In developing countries, an increase of IBD incidence and prevalence occurred over the last 30 years, when Westernized lifestyle and dietary habits were adopted [2, 3]. A contribution of environmental factors to the rapid rise of IBD incidence is highly probable, considering that genetic susceptibility has been present in human beings since thousands of years without significant changes in this short period. Environmental risk factors, which affect the course of IBD are currently studied with epidemiological approaches and some influencing factors, such as breastfeeding, diet or antibiotic use have been identified [4–6].

Diet is widely considered as a key environmental factor. Dietary changes affect the composition of the gut microbiome, which may influence mechanisms of immunological tolerance [7, 8]. Western diet is mainly composed of high-sugar, low-fiber, animal-protein and fat, but low ingestion of vegetables [9, 10]. Epidemiological data suggest, that a ‘Westernization’ of the diet may induced mucosal inflammation in susceptible individuals and may act as promoter in the pathogenesis of IBD [11]. Recent data demonstrated a positive correlation between pro-inflammatory and ultra-processed food intake and the risk of developing IBD [12, 13].

The reason why a dysbiosis of the intestinal microbiota promotes the development of IBD is not fully understood. A plausible explanation is the increase in potentially pathogenic bacterial species combined with a decrease in protective bacteria, resulting a disruption of local immune homeostasis, increased mucosal permeability and loss of immune tolerance [14].

Literature data is scarce regarding the association between IBD and dietary behavior in the childhood period, considering that a subclinical intestinal inflammation can exist a long time before the outbreak of the disease [7]. In addition, not much is known about overweight in childhood and its influence on development of IBD or disease activity. Previous work implies an association between overweight and more severe disease activity in children with IBD [15].

Better knowledge regarding dietary habits would be of utmost importance for patients to reduce flare-ups, but also to prevent the development of IBD.

Likewise, breastfeeding and mode of delivery are considered as an important factor regarding the composition of the microbiome in the early childhood period and have been associated with the development of IBD [16–18]. A review of Ananthakrishnan et al. [19] outlined a protective effect of being breastfed for IBD. Concerning the mode of delivery as a risk factor for IBD, findings in literature are inconsistent. While various studies postulated Caesarean section (C-section) as a risk factor for IBD, a population-based study of Bernstein et al. found no association between IBD and mode of delivery [16, 20, 21].

Physical activity levels have been associated as a protective factor regarding the risk of IBD due to a reduction of systemic inflammation [22–26]. Physical activity has various effects on immunomodulatory processes and affects the balance of inflammatory and anti-inflammatory mechanisms [27, 28]. Investigations showed that incidence of autoimmune diseases such as rheumatoid arthritis, multiple sclerosis or psoriasis is higher in patients less engaged in physical activity [29].

According to present experience we have a lack of knowledge concerning nutrition and diet as well as physical activities among patient with IBD and their potential impact on disease development. Increased knowledge of early-life risk factors of IBD ensures better prevention of disease and may reduce the number of IBD patients.

Main goal of this study is to figure out the impact of lifestyle factors such as dietary habits, weight status and physical activity on IBD in Swiss patients, especially regarding childhood period and adolescence.

## Materials and methods

### Study design

Prospectively obtained data from patients of the Swiss IBD cohort study (SIBDCS), a nationwide cohort study funded by the Swiss National Science Foundation were analyzed. In addition to patients diagnosed with IBD, the cohort has included two related cohorts of patient’s friends and mothers to evaluate the impact of various environmental factors on IBD risk. Clinical and treatment data have been prospectively captured with a yearly follow-up and entered into a database since the establishment of the cohort in 2006. Purposes and methodology of SIBDC have been described elsewhere [30, 31].

Questionnaires regarding various environmental factors were distributed to IBD patients (n = 2294) between December 2015 and October 2016 in Swiss national languages. Questionnaires returned until January 2018 were included in this study. In addition, identical questionnaires were sent out to up to three matched childhood friends to form a control group (friend’s cohort), which had been exposed to a similar environment as the patients. To double-check information relating to patient’s early childhood period, a questionnaire addressing the patients’ mothers was sent out. Concerning potential influencing factors on IBD such as physical activity, overweight and obesity we focused on childhood period and beginning of adulthood.

Our intention was to obtain information from the point of birth to the age of about 4 years. In the interest of simplification, we defined this period in our questionnaires ‘first years of life’.

We further categorized sugary foods into sugary drinks (Coca-Cola, Fanta etc.), artificial sugary (jelly-babies, bonbons) and natural sugary (e.g. chocolate).

### Statistical analysis

Statistical analyses were performed using the Version 16.0 of the Stata software (College Station, TX 77,845 USA) with univariate, bivariate and multivariate analysis. Univariate analyses were performed to summarize the variables and multivariate analysis was used to evaluate the impact of some explanatory variables on dietary habits. The qualitative variables were summarized with percentages and the quantitative variables with the mean and the standard deviation when they were normally distributed or with the median and the interquartile range when they were not. For bivariate analyses, the Chi-square test was performed to study the relationship between the categorical variables. We performed the Student test to compare the means between the groups when the variables are normally distributed. The Wilcoxon–Mann–Whitney test was used to compare the means when the variables are not normally distributed.

## Results

### Clinical characteristics of the study population

Out of 2294 questionnaires handed out to SIBDC patients, 1111 were sent back (response rate: 48.4%). Additional information from mother questionnaires were available for 305 out of 1111 responding patients (response rate: 27.5%). In addition, we obtained 225 questionnaires from at least one patients’ friend (Response rate: 20.3%). A total of 352 friends’ questionnaires were received.

Divided in subtypes, we received 610 questionnaires from patients with CD and 468 with UC/IC. For further clinical and epidemiological parameters of CD and UC/IC we refer to Table 1.

**Table 1:** Clinical characteristics of the study population

	CD (n = 610)	UC/IC (n = 468)	Controls (n = 365)	*p*-value
Gender [n (%)]				
Male	271 (44.4%)	232 (49.6)	130 (35.6)	**0.001**
Female	339 (55.6%)	236 (50.4)	235 (64.4)	
Age [y]	48.5, 15.5	50, 14	36, 9	**0.0001**
(Mean, SD, range)	18–87	19–85	18–75	
BMI [kg/m^2^]	23.6, 3.6	24.5, 3.7	21.3, 3.1	0.224
(Mean, SD, range)	18–30	18–31.5	18–25	
Age at diagnosis [y]	31, 14	34, 14		**0.0001**
(Mean, SD, range)	1–75	8–78		
Disease duration [y]	18, 11	16, 10		**0.0034**
(Mean, SD, range)	0–53	0–52		
CDAI	20, 41			
(Median, IQR, range)	0–230			
MTWAI		1, 3		
(Median, IQR, range)		0–17		
Smoking status at diagnosis [n (%)]				
Non-smoker	350 (57.7)	371 (75.7)		**0.001**
Smoker	235 (38.7)	93 (19)		
Unknown	22 (3.6)	26 (5.3)		
Current smoking status [n (%)]				
Non-smoker	453 (74.6)	319 (85.5)		**0.001**
Smoker	151 (24.9)	66 (13.5)		
Unknown	3 (0.5)	5 (1)		
Therapy history (ever treated with) [n (%)]				
5-ASA	369 (60.8)	471 (96.0)		**0.001**
Steroids	528 (87)	398 (81)		**0.009**
Immunomodulators	492 (81.1)	300 (61.2)		**0.001**
Anti-TNF	384 (63.3)	169 (34.5)		**0.001**
EIM [n (%)]				
Any	380 (62.6)	219 (44.7)		**0.001**
Arthritis	330 (54.4)	173 (35.3)		**0.001**
Uveitis/Iritis	79 (13)	30 (6.1)		**0.001**
Pyoderma gangraenosum	8 (1.3)	9 (1.8)		0.489
Erythema nodosum	62 (10.2)	15 (3.1)		**0.001**
Aphtous/Oral ulcers	93 (15.3)	25 (5.1)		**0.001**
Ankylosing spondylitis	38 (6.3)	16 (3.3)		**0.023**
PSC	8 (1.3)	15 (3.1)		**0.045**
Complications [n (%)]				
Perianal fistula	164 (27)	–		
Other fistula	111 (18.3)			
Abscess	137 (22.6)			
Stenosis	264 (43.5)			
Surgery history [n (%)]				
Any	313 (51.6)	53 (10.8)		**0.001**
Intestinal surgery	258 (42.5)	46 (9.4)		**0.001**
Fistulas or abscess surgery	147 (24.2)	10 (2)		**0.001**

All tests were two-sided, with a *P* value of less than 0.05 considered to indicate statistical significance. These results are highlighted in bold

### Physical activity during childhood and beginning of adulthood

For the period of childhood to the begin of adulthood, the report for 257 (42.1%) patients with CD and 181 (38.7%) patients with UC/IC presented no regular activity, whereas only 107 (29.3%) persons from the control group did not practice sport regularly (*p* = 0.001) (Table 2). More individuals from the control group (179, 49%) practiced sport alone or in a club, than patients with CD (248, 40.7%) or UC/IC (197, 42.1%, *p* = 0.032). There was a trend that more UC/IC patients (43, 9.2%) practiced frequently high-level sport than CD patients (34, 5.6%, *p* = 0.051). Regarding practicing endurance sport no difference between IBD patients and individuals from the control group was observed.

**Table 2:** Physical activity during childhood and beginning of adulthood

	CD (n = 610)	UC/IC (n = 468)	Controls (n = 365)	*p*-value CD versus UC/IC	*p*-value IBD versus controls
Regular practice of sport [n (%)]					
Yes	353 (57.9)	287 (61.3)	258 (70.7)	0.252	**0.001**
No	257 (42.1)	181 (38.7)	107 (29.3)		
Regular practice of sport alone or in a club [n (%)]					
Yes	248 (40.7)	197 (42.1)	179 (49)	0.635	**0.032**
No	362 (59.3)	271 (57.9)	186 (51)		
Practice sport more than people in the same age [n (%)]					
Yes	65 (10.7)	49 (10.5)	51 (14)	0.922	0.210
No	545 (89.3)	419 (89.5)	314 (86)		
High level practice of sport frequently [n (%)]					
Yes	34 (5.6)	43 (9.2)	32 (8.8)	0.051	0.491
No	576 (94.4)	425 (90.8)	333 (91.2)		
Endurance sport [n (%)]					
Yes	2 (0.3)	3 (0.6)	4 (1.1)	0.453	0.337
No	608 (99.7)	465 (99.4)	361 (98.9)		

All tests were two-sided, with a *P* value of less than 0.05 considered to indicate statistical significance. These results are highlighted in bold

### Breastfeeding and mode of birth

A higher number of individuals from the control group (243, 68.1%) had been breastfed as compared to patients with IBD, especially with IC/UC (279, 59.9%, *p* = 0.002) (Table 3). Significantly more persons from the control group reported that their baby bottles and teats had been sterilized (139, 43.8%) compared to IBD patients (CD: 172, 32.6%, UC/IC: 127, 31.4%). The number of individuals from the control group born by C-section (50, 13.7%) was significantly higher than in the IBD group (*p* = 0.001). Premature birth has significantly more often occurred in the CD fraction (61, 10.2%) than in the control group (28, 7.8%).

**Table 3:** Breastfeeding and mode of birth

	CD (n = 610)	UC/IC (n = 468)	Controls (n = 365)	*p*-value CD versus UC/IC	*p*-value IBD versus controls
Have you been breastfed [n (%)]					
Yes	398 (65.7)	279 (59.9)	243 (68.1)	**0.033**	**0.002**
No	105 (17.3)	112 (24)	81 (22.7)		
Don’t know	103 (17)	74 (15.9)	32 (9)		
Missing	4	3	9		
Sterilization of baby bottles and teats [n (%)]					
Yes	172 (32.6)	127 (31.4)	139 (43.8)	0.284	**0.001**
No	62 (11.7)	35 (8.6)	49 (15.5)		
Don’t know	293 (55.5)	243 (60)	129 (40.7)		
Missing	82	63	48		
Sterilization frequency of baby bottles and teats [n (%)]					
After each meal	45 (11.5)	30 (9.7)	40 (17.5)	0.781	**0.021**
Once a day	45 (11.5)	40 (13)	42 (18.4)		
Once per week	19 (5)	17 (5.5)	12 (5.3)		
Don’t know	283 (72)	221 (71.8)	134 (58.8)		
Missing	218	160	137		
Mode of birth					
Natural childbirth [n (%)]	495 (81.2)	389 (83.1)	291 (79.3)	0.403	0.446
Caesarean-section [n (%)]	49 (8)	28 (6)	50 (13.7)	0.195	**0.001**
Forceps delivery [n (%)]	14 (2.3)	13 (2.8)	6 (1.6)	0.615	0.554
Delivery with cupping [n (%)]	13 (2.1)	17 (3.6)	10 (2.7)	0.137	0.330
I don’t know [n (%)]	37 (6.1)	22 (4.7)	7 (1.9)		
Are you born at term [n (%)]					
Yes	423 (70.7)	349 (75.2)	268 (74.4)	0.111	**0.017**
Premature birth	61 (10.2)	30 (6.5)	28 (7.8)		
Birth triggered after the term	37 (6.2)	19 (4.1)	32 (8.9)		
Don’t know	75 (12.5)	64 (13.8)	32 (9)		
Missing	14	6	5		

All tests were two-sided, with a *P* value of less than 0.05 considered to indicate statistical significance. These results are highlighted in bold

### Dietary habits

Significantly more individuals from the control group (187, 51.2%) reported they had drunk packet cow milk from the supermarket during the first years of life than IBD patients (*p* = 0.001) (Table 4). On the other hand, more patients suffering from IBD (CD: 158, 25.9%, UC/IC: 118, 25.2%) drank cow milk directly from the farm during the first years of life than exponents from the control group (62, 17%, *p* = 0.003). Concerning the kind of milk there were slightly more individuals from the controls (53, 14.5%) who drank semi-skimmed milk during the first years of life than patients diagnosed with IBD (CD: 57, 9.3%, UC/IC: 49, 10.5%). Comparing other kind of milk between controls and IBD patients showed no significant difference.

**Table 4:** Nutritional habits during the first years of life

	CD (n = 610)	UC/IC (n = 468)	Controls (n = 365)	*p*-value CD versus UC/IC	*p*-value IBD versus controls
Did you regularly drink cow’s milk during the first years of your life					
Packed (super market) [n (%)]	208 (34.1)	145 (31)	187 (51.2)	0.280	**0.001**
Directly from the farm [n (%)]	158 (25.9)	118 (25.2)	62 (17)	0.798	**0.003**
Yes one or the other [n (%)]	92 (15.1)	81 (17.3)	58 (15.9)	0.324	0.612
No [n (%)]	38 (6.2)	32 (6.8)	18 (4.9)	0.688	0.514
I don’t know [n (%)]	115 (18.8)	94 (20.1)	39 (10.7)		
What kind of milk did you drink in the first years					
Whole milk 3.5% fat [n (%)]	305 (50)	249 (53.2)	198 (54.3)	0.297	0.371
Semi-skimmed milk 1.5% fat [n (%)]	57 (9.3)	49 (10.5)	53 (14.5)	0.538	**0.04**
Skimmed milk < 0.3% [n (%)]	5 (0.8)	4 (0.9)	4 (1.1)	0.950	0.900
Raw milk [n (%)]	88 (14.4)	78 (16.7)	51 (14)	0.312	0.478
Milk without lactose [n (%)]	3 (0.5)	0	0	0.129	0.128
Soy milk [n (%)]	3 (0.5)	2 (0.4)	4 (1.1)	0.877	0.412
I don’t know [n (%)]	158 (26)	110 (23.8)	69 (19.1)		
Did you tolerate the milk well					
Yes [n (%)]	398 (65.3)	343 (73.3)	279 (76.4)	**0.005**	**0.001**
No [n (%)]	131 (21.5)	74 (15.8)	62 (17)	**0.019**	**0.041**
I don’t know [n (%)]	73 (12)	44 (9.4)	17 (4.7)		
What are the symptoms that you had after consuming milk					
Bloating [n (%)]	60 (9.8)	37 (7.9)	32 (8.8)	0.272	0.541
Pain [n (%)]	29 (4.7)	17 (3.6)	11 (3)	0.366	0.367
Diarrhea [n (%)]	63 (10.3)	21 (4.5)	19 (5.2)	**0.001**	**0.001**
Nausea [n (%)]	30 (4.9)	15 (3.2)	17 (4.7)	0.163	0.360
Other [n (%)]	83 (13.6)	62 (13.3)	34 (9.3)	0.864	0.115
I don’t know [n (%)]	90 (14.8)	64 (13.7)	24 (6.6)		

All tests were two-sided, with a *P* value of less than 0.05 considered to indicate statistical significance. These results are highlighted in bold

279 out of 365 controls (76.4%) reported they had tolerated the milk well during the first years of life, while significant less patients suffering from IBD tolerated the milk well (*p* = 0.001). Comparing the subgroups of IBD there were significant more patients with CD (131, 21.5%) complaining about symptoms after drinking milk in the first years of life than patients with UC/IC (74, 15.8%, *p* = 0.019). When investigating the several symptoms in more detail a higher number of CD patients (63, 10.3%) complained about diarrhea during the first years of life after drinking milk than patients suffering from UC (21, 4.5%, *p* = 0.001) and controls (19, 5.2%).

When observing the analysis of eating behavior of sugary foods until the age of 18 years no significant differences between IBD patients and controls stood out (Table 5). Regardless of the stage of life more patients diagnosed with IBD (CD: 24, 3.9%, UC/IC: 17, 3.6%) reported to feed their selves on a vegetarian basis than individuals from the control group (Table 6). In addition, more patients diagnosed with CD (88, 14.4%) consumed a meat-rich diet than patients with UC/IC (40, 8.6%, *p* = 0.003) and controls (39, 10.7%) Considering other special diets such as gluten free or vegan alimentation no significant difference between IBD patients and controls has been observed.

**Table 5:** Sugary foods until the age of 18

	CD (n = 610)	UC/IC (n = 468)	Controls (n = 365)	*p*-value CD versus UC/IC	*p*-value IBD versus controls
Sugary drinks [n (%)]					
More than other children	24 (4.1)	21 (4.6)	13 (3.6)	0.716	0.906
In average like other children	285 (48.1)	229 (50)	176 (48.8)		
Less than other children	283 (47.8)	208 (45.4)	172 (47.7)		
Missing	18	10	4		
Eating artificial sugary [n (%)]					
More than other children	33 (5.7)	25 (5.5)	18 (5)	0.765	0.278
In average like other children	280 (48)	228 (50)	203 (56.2)		
Less than other children	269 (46.1)	203 (44.5)	140 (38.8)		
Missing	27	12	4		
Eating natural sugary [n (%)]					
More than other children	54 (9.2)	45 (9.8)	36 (9.9)	0.720	0.427
In average like other children	406 (69.1)	324 (70.4)	266 (73.5)		
Less than other children	128 (21.8)	91 (19.8)	60 (16.6)		
Missing	22	8	1

**Table 6:** Eating habits or special diets

	CD (n = 610)	UC/IC (n = 468)	Controls (n = 365)	*p*-value CD versus UC/IC	*p*-value IBD versus controls
Consuming food like the average [n (%)]					
Yes	504 (82.6)	401 (85.7)	303 (83.1)	0.175	0.369
No	106 (17.4)	67 (14.3)	62 (17)		
Vegetarian alimentation [n (%)]					
Yes	24 (3.9)	17 (3.6)	30 (8.2)	0.797	**0.003**
No	586 (96.1)	451 (96.4)	335 (91.8)		
Vegan alimentation [n (%)]					
Yes	2 (0.3)	4 (0.9)	4 (1.0)	0.249	0.329
No	608 (99.7)	464 (99.1)	361 (98.9)		
Gluten free [n (%)]					
Yes	12 (2)	10 (2.1)	5 (1.4)	0.845	0.701
No	598 (98)	458 (98)	360 (98.6)		
Low-lactose or lactose free [n (%)]					
Yes	42 (6.9)	27 (5.8)	16 (4.4)	0.458	0.273
No	568 (93.1)	441 (94.2)	349 (95.6)		
Fastfood or finished products more than 3 times a week [n (%)]					
Yes	8 (1.3)	8 (1.7)	3 (0.8)	0.592	0.537
No	602 (98.7)	460 (98.3)	362 (99.2)		
Meat-rich diet [n (%)]					
Yes	88 (14.4)	40 (8.6)	39 (10.7)	**0.003**	**0.009**
No	522 (85.6)	428 (91.5)	326 (89.3)		

All tests were two-sided, with a *P* value of less than 0.05 considered to indicate statistical significance. These results are highlighted in bold

### Overweight or obesity during childhood and beginning of adulthood

One hundred and four out of 610 patients with CD (17.2%) reported they had insufficient weight or were very thin during childhood compared to children of their age (Table 7). Compared with the controls (35, 9.7%) and patients diagnosed with UC/IC (36, 7.7%, *p* = 0.027) significantly more patients with CD suffered from overweight during childhood (77, 12.8%, *p* = 0.012).

**Table 7:** Overweight or obesity during childhood and beginning of adulthood compared to children of their age

	CD (n = 610)	UC/IC (n = 468)	Controls (n = 365)	*p*-value CD versus UC/IC	*p*-value IBD versus controls
During childhood [n (%)]					
Insufficient weight/very thin	104 (17.2)	77 (16.5)	54 (14.9)	**0.027**	**0.012**
Weight in the average	410 (67.9)	341 (73.2)	268 (74)		
Overweight, round, coated	77 (12.8)	36 (7.7)	35 (9.7)		
Obese	5 (0.8)	0	5 (1.4)		
I don’t know	7 (1.2)	9 (2)	0		
Missing	7	6	3		
At the beginning of adulthood [n (%)]					
Insufficient weight/very thin	102 (16.9)	56 (12.1)	35 (9.7)	**0.027**	**0.014**
Weight in the average	441 (73.3)	376 (81)	287 (79.3)		
Overweight, round, coated	53 (8.8)	28 (6)	37 (10.2)		
Obese	4 (1)	2 (0.4)	3 (0.8)		
Missing	8	6	3		

All tests were two-sided, with a *P* value of less than 0.05 considered to indicate statistical significance. These results are highlighted in bold

Similarly, at beginning of adulthood, more CD patients (102, 16.9%) had insufficient weight or were very thin than UC/IC patients (56, 12.1%, *p* = 0.027) or controls (35, 9.7%). In contrary to the childhood period, controls (37, 10.2%) tended to have more overweight than patients with CD (58, 8.8%) and UC/IC (28, 6%).

## Discussion

Based on 1111 questionnaires of IBD patients we aimed to identify associations between environmental factors and the development of IBD. Our data confirm physical activity as a protective factor for IBD. Consuming meat-rich diet was associated with developing CD. On the other hand, no correlation between intake of sugar and development of IBD was observed. Overweight during childhood was associated for CD, but not for UC. Underweight during childhood and adulthood was associated with both, CD and UC/IC.

Our results support the hypothesis of a protective effect of physical activity regarding the development of IBD. Patients diagnosed with IBD reported to be less physically active during childhood and beginning of adulthood than persons from the control group. These findings are in line with the result of a review of meta-analyses recently published, that demonstrated a protective effect of physical activity regarding the development of CD [32]. On the other hand, a Danish prospective cohort study reported no association between physical activity and risk of IBD [33]. A possible explanation for this discrepancy to our result is, that the Danish study did not investigate the association between timing in life of physical activity and risk of IBD. The influence of physical activity on the onset of IBD is still unclear and evidence in literature is scarce. There is consensus in literature that physical activity has an impact on various aspects of the immune system and autoimmune diseases [29]. An investigation of Steensberg et al. [34] implied that sporting activity induces a shift in the Th1/Th2 balance to a decrease in Th1 cells. Th1 is responsible for secretion of proinflammatory cytokines as IL-1, IL-2, IL-6 and IL-8, whereas anti-inflammatory cytokines as IL-4, IL-10 and IL-13 are secreted by Th2 cells. Thus, the balance between proinflammatory and anti-inflammatory mechanisms is highly affected by the Th1/Th2 cells ratio and responsible for the types of immune responses that patients develop [35]. Considering other diseases driven by autoimmune processes such as rheumatoid arthritis, multiple sclerosis or psoriasis, studies have shown an increased incidence in patients less engaged in physical activity [29].

Lack of exercise may result in obesity, what is assumed to be a cause for a chronic low-grade inflammation in humans [36]. It is explained, amongst others, by a predominance of pro-inflammatory macrophages in mesenteric visceral adipose tissue, that is responsible for secretion of various inflammatory cytokines, including IL-1 and TNF [37]. Kugathasan et al. [38] demonstrated that about 9–10% of children with CD and 20–34% of children with UC had an increased BMI above the 85th percentile at diagnosis. In contrary, a recently published study demonstrated no worsened disease activity 1 year after diagnosis of IBD of overweight children compared with normal weight children [39]. Our patients with CD reported to be more often overweight compared to children of their age during childhood. Both, CD and UC patients reported to be underweighted compared to children of their age during childhood and adulthood. A recently published meta-analysis reported an positive association between underweight and the onset of CD, but not for UC [40].

The impact of breastfeeding on the onset of IBD is also under debate. Previous trials demonstrated that alteration in the composition of the microbiota disrupts microbial mediated mechanisms of immunological tolerance [7, 8]. Human milk contains, among others, oligosaccharides with prebiotic effects including growth of Bifidobacteria which may affect the intestinal flora and influence the risk of IBD [41]. Our data support the assumption of a protective effect of breastfeeding, especially considering the onset of UC/IC. Furthermore, our attempt was to obtain information regarding breast milk substitution and identify bottle-feeding and frequency of sterilization of baby bottles and teats as possible risk factors for IBD. Investigations suggest a positive association of breastfeeding and development of IBD, but some controversy remains in literature [6, 17, 42]. Unfortunately, most of patients, friends and mothers had to answer the questions in this part with ‘don’t know’, whereby the data is not conclusive. Therefore, additional investigations are warranted.

Our findings do not support the thesis that C-section enhances the risk of IBD, as more persons from the control group reported to be born via C-section. Confounders may distort the result and the findings have to be treated with caution. A population-based analysis of Bernstein reported no association of C-section and IBD [21]. On the other hand a meta-analysis by Li et al. [16] indicated an increased risk for CD but not for UC after Cesarean delivery.

Interestingly, significantly more patients with CD than persons from the control group reported to be born prematurely. Although perinatal mortality has been considerably reduced during the past years, prematurely born infants are still liable to a higher mortality and morbidity rate in comparison with infants born at term [43]. These finding are in line with a work of Sonntag et al. [44] that identified preterm birth as a risk factor for CD.

Investigations suggest that diet plays an important role in IBD. Dietary habits have been changed in the western world during recent decades and cause alterations in the composition of the gut microbiota, that may result in aberrant intestinal immune response [45, 46]. A subclinical intestinal inflammation can be present long before occurrence of the first IBD symptoms [7]. For this reason, it is of utmost importance to identify risk factors that affected patients before the clinically manifest disease. Our data indicate that more people from the control group consumed semi-skimmed milk (1.5% fat) during the first years of life than patients with IBD. It has been shown that high fat intakes cause an accumulation of secondary bile acids, what is responsible for reduction in growths of *Firmicutes phyla* and *Bacteroidetes*, both associated with IBD-like dysbiosis [47]. On the other hand, a prospective study of Ananthakrishnan et al. [48] indicated no association of fat consumption and IBD in women. Interestingly, patients diagnosed with CD reported to have had a milk intolerance during their first years of life, clinically characterized by diarrhea. A positive correlation between IBD and lactose intolerance is well established in literature, but evidence is scarce that a milk intolerance might be already present during the first years of life and a long time before the diagnosis of the disease [49]. The association between sugar intake and risk of IBD remains controversial. Several studies showed a negative effect of consuming sugary food on the onset of IBD [50–52]. In contrast to these findings, a large prospective study could not identify sugar intake as a risk factor for developing UC [53]. Our data suggest no connection between IBD and eating of neither artificial nor natural sugary until the age of 18. Literature is very scarce regarding dietary habits before 18 and development of IBD. Ananthakrishnan et al. [54] published an investigation regarding high school diet and risk of CD and UC, which demonstrated no association between intake of carbohydrates and onset of IBD.

Previous studies have shown a correlation between diet rich in animal protein and development of IBD [55, 56]. Our data support these findings and we report that statistically significant more CD patients consume meat-rich diet in comparison to the control group. A plausible explanation is that high intake of protein result in increased production of potentially toxic bacterial metabolites, what may lead to an impaired epithelial repair process [57]. As well, our data implicate a reduced consumption of vegetarian alimentation in IBD patients, what confirms the assumption that diets in high animal proteins lead to increased risk of IBD.

Our study has strengths as well as weaknesses. A strength is the large amount of IBD patients in our cohort with in total 1111 returned questionnaires. Furthermore, we aimed to reduce the ‘recall bias’ due to 305 returned questionnaires from patients’ mothers.

One limitation is the low overall return of questionnaires. The low response rate of the patient’s friends resulted in a small control group compared to the large number of IBD patients. Probably it was more difficult than expected for patients to reach childhood friends who grew up in a similar environment. Even though a high response rate is preferable, previous studies could demonstrate that there is no evidence of more accurate measurement in surveys with higher response rates [58, 59].

Furthermore, we are aware that our methodology is of risk of ‘recall bias’. Even though the data of patient’s mothers reduce the ‘recall bias’, incorrect memories of behavior during childhood may exist. To confirm our findings further prospective randomised trials are needed. As well, our analyses were not matched for potential confounders. We performed a multivariate analysis included all the investigated risk factors matched for sex, age and smoking status. As there was no significant difference in the results to our published data, we decided to exclude the multivariate analysis.

In conclusion, our data demonstrate the possibility that lifestyle factors such as physical activity, dietary habits and weight status affect the onset of IBD and may play a crucial role in preventing IBD (Table 8). This study indicates that education and prevention strategies may reduce the increasing incidence of patients with inflammatory bowel disease.

**Table 8:** Overview of the most important findings regarding effects in regard to the development of CD and UC/IC (Statistical analyses are seen in Tables 2, 3, 4, 5, 6, 7)

Environmental factor	CD	UC/IC
Physical activity	Protective	Protective
Breastfeeding	No significant effect	Protective
Premature birth	Negative effect	No significant effect
Sugar intake	No significant effect	No significant effect
Meat-rich diet	Negative effect	No significant effect
Vegan-/vegetarian alimentation, gluten free diet,	No conclusive data	No conclusive data
Overweight during childhood	Negative effect	No significant effect
Underweight during childhood and adulthood	Negative effect	Risk factor

## Data Availability

The datasets used and analysed during the current study are available from the corresponding author on reasonable request.

## Abbreviations

IBD: Inflammatory bowel disease
CD: Crohn’s disease
C-section: Caesarean section
EF: Environmental factors
UC: Ulcerative colitis
IC: Indeterminate colitis
SIBDCS: Swiss inflammatory bowel disease cohort study
EIM: Extraintestinal manifestation
PSC: Primary sclerosing cholangitis
